# What challenges hamper Kenyan family physicians in pursuing their family medicine mandate? A qualitative study among family physicians and their colleagues

**DOI:** 10.1186/1471-2296-13-32

**Published:** 2012-04-26

**Authors:** Chiel TM van der Voort, Geraldine van Kasteren, Patrick Chege, Geert-Jan Dinant

**Affiliations:** 1 Faculty of Health, Medicine and Life Sciences, School of Health Professions Education, Maastricht University, Maastricht, The Netherlands; 2 Division of Family Medicine, Moi University, P.O. Box 4606, 030100, Eldoret, Kenya; 3 School for Public Health and Primary Care (Caphri), Department of General Practice, Maastricht University, P.O. Box 616, 6200 MD, Maastricht, The Netherlands

## Abstract

**Background:**

Since 2005, Kenyan medical universities have been training general practitioners, providing them with clinical, management, teaching and research skills, in order to enhance access to and quality of health care services for the Kenyan population. This study assesses what expectations family physicians, colleagues of family physicians and policy makers have of family medicine, what expectations family physicians live up to and which challenges they face.

**Methods:**

Family physicians were observed and interviewed about their expectations and challenges concerning family medicine. Expectations among their colleagues were assessed through focus group discussions. Policy makers’ expectations were assessed by analysing the governmental policy on family medicine and a university’s curriculum.

**Results:**

Roles perceived for and performed by family physicians included providing comprehensive care, health care management, teaching, and to a lesser extent community outreach and performing research. Challenges faced by family physicians were being posted in situations where they are regarded as just another type of specialist, lack of awareness of the roles of family physicians among colleagues, lack of time, lack of funds and inadequate training.

**Conclusions:**

The ministry’s posting policy has to be improved to ensure that family physicians have a chance to perform their intended roles. Creating an environment in which family physicians can function best requires more effort to enlighten other players in the health care system, like colleagues and policy makers, about the roles of family physicians.

## Background

Primary Health Care (PHC) established its role during the Alma-Ata conference in the USSR in 1978 [1]. Since then, many countries have established PHC in their health care systems [2]. Within PHC, there is an important role for the family physician (FP) [2-4].

Starfield and Shi have shown that in industrialised countries, a stronger PHC sector results in better health outcomes at lower costs [5]. Improvements in health outcomes are especially profound in early life. Increasing the ratio of FPs to other medical specialists is acknowledged to have a beneficial influence on the health of a population [6,7]. Efforts to introduce FPs in the health care systems have also been made in Sub-Saharan Africa (SSA). After countries like South Africa and Nigeria, Kenya established a family medicine residency programme (hereafter: ‘the programme’) [8]. Other countries, including Tanzania, Uganda, Rwanda and Ethiopia, are in the process of extending their PHC services through similar programmes [8-11]. To ensure appropriate physicians for local populations, several SSA countries have developed varying concepts of PHC workers. In Ethiopia, PHC workers are referred to as health extension workers, who upon completion of a 1-year training course deliver care comprising disease prevention and control, hygiene and sanitation, family health services and health education [10]. In an attempt to increase conformity among SSA countries, a panel of 40 experts reached consensus in 2006 on the key principles of family medicine. The panel consisted of graduated FPs, physicians participating in family medicine training programmes and individuals responsible for training FPs [12].

The Master of Medicine degree in Family Medicine (MMed-FM) offered at Moi University in Kenya endeavours to provide FPs with clinical, management, teaching and research skills [8]. It was created in response to the low accessibility of health care services for the majority of the 39.4 million Kenyan inhabitants, about 80% of whom live in rural areas [13]. After 3 years of training, FPs are expected to perform a wide range of tasks, specified in 6 objectives: (1) providing and coordinating continuous, comprehensive and cost-effective health care to individuals, families and communities, (2) engaging in lifelong learning using various modalities and being able to evaluate their own performance, (3) carrying out health-related research and appropriately using information from research, (4) effectively teaching members of the health care team, patients and the community, (5) practicing in an ethical manner, and (6) managing health care resources [14]. So far, 13 FPs have graduated and acquired positions within the Kenyan health care system [Van Kasteren & Van den Hombergh, unpublished data].

To improve accessibility and equitable provision of quality health care, Kenya needs a sufficient number of FPs. However, training FPs is merely a start. In order to intensify returns, FPs need to be sufficiently facilitated, as well as integrated in the existing health care system. Health outcomes are likely to depend on cooperation between (1) FPs, (2) colleagues working in the same healthcare setting (eg. medical doctors, clinical officers, nurses and lab technicians, referred to below as ‘colleagues’) and (3) the government as policy makers. These three parties are referred to below as ‘the 3 players’. Ideally, research should also include assessments of patients, private general practitioners and more distant colleagues (eg. colleagues in different health care settings, whom, the FPs consult or refer patients to). In this early stage, however, we decided that the impact of the programme is as yet too small for us to be able to acquire appropriate data from these relevant players. Thus, in the context of this study, an integrated FP is defined as one who does what the 3 players expect from him/her. In other words, perfect integration of FPs is achieved when the expectations of the 3 players are in line with each other. By contrast, conflicting expectations can be a result of various types of constraints experienced by FPs. The research question we used as a starting point is: how well are FPs integrated in the existing health care system? To answer this question, we conducted a qualitative study involving a review of key documents, interviews and focus group discussions (FGDs) with FPs and their colleagues, as well as observation of FPs at work.

## Methods

The expectations policy makers had of FPs were assessed by analysing the following documents: the governmental policy on family medicine (hereafter: the policy) [15], the curriculum of the MMed-FM degree (hereafter: the curriculum) [14], the proposed strategy for family medicine (hereafter: the strategy) [16] and the roles of FPs as defined by the Committee of Family Medicine [17]. The Kenyan constitution, approved in 2010, [18] was reviewed to get a broader idea of the political climate. On the basis of these documents, we identified the themes to be addressed during the interviews and the FGDs.

To be eligible FPs had to be graduated with the MMed-FM degree at Moi University in Eldoret, Kenya. The thirteen eligible FPs were invited by phone and agreed to participate. They were then visited at the institutions where they had been posted. The FPs were based in rural district hospitals in Central Province, North Rift Valley and Western Province in Kenya. Information on the study was given to them both verbally and in writing. All FPs confirmed their willingness to participate in the study by filling in an informed consent form. The semi-structured interviews were conducted in English by the principal investigator and took 50 to 90 minutes. They included questions on the FP’s decisions to join the family medicine programme, their ideas of what family medicine and being an FP entails, the roles the FP actually performs and the challenges FPs face while performing their roles [see Additional file 1]. After nine interviews with FPs, data saturation appeared to have been reached, as no new views came forward from the sixth interview onward. Hence, four potential participants were not interviewed.

Based on their location within a few hours reach of Nairobi, three FPs were selected for observation. Prior to their selection, no knowledge was gathered about these FPs and their settings other than their geographical location. We ensured that the medical superintendent of the hospital agreed with the principal investigator’s stay. In two of the three cases, the FP happened to be the medical superintendent himself. Observations lasted three to five working days per FP. Notes were taken throughout the observations to capture what expectations were fulfilled by the FPs.

Colleagues of the FP at these three institutions were invited to participate in an FGD. These colleagues had to be health care workers employed in the same health care setting as the participating FP. Informal invitations were extended verbally to colleagues selected by the principal investigator. Sometimes the invitation was based on the principal investigator’s perception that this particular colleague would be interesting and would be inclined to air his or her opinions. In addition to these invitations, other colleagues or groups of colleagues were invited. The intention was to select a diverse and outspoken group of colleagues. In total, approximately 25 colleagues were invited, eleven of whom took part in the FGDs (two to five per FGD, five nurses, two clinical officers who had completed a 3-year programme in clinical medicine, two medical officer interns who were in the process of becoming a doctor, one physical therapist, one lab technician).

The FGDs were held while the participants were on duty, and on one occasion a nurse had to leave the FGD for some minutes, after which she rejoined. It happened twice that participants left the FGD for some minutes to answer their phones. The FGDs were held in English, took 30 minutes to one hour and were moderated by the principal investigator. Questions were asked about the situation in the hospital, what they perceived family medicine to be, what colleagues expected from FPs and whether expectations were fulfilled [see Additional file 2]. All interviews and focus group meetings were recorded and transcribed, facilitated by Dragon NaturallySpeaking software. The key documents, observation notes and answers obtained during the interviews and FGDs were coded, categorised and analysed thematically using qualitative analysis software (NVivo). Ethical approval for the study was obtained from the designated board at Moi University. The data was gathered from May to July 2011.

## Results

The interviews and FGDs proved most informative. The observations provided some contextual information and opportunities to invite appropriate participants for the FGDs. Although the observations as such added only marginally to the findings, they are likely to have enabled the FGDs to succeed. Three main topics emerged from the data obtained: ambition to become an FP, roles perceived for and fulfilled by FPs and challenges to FPs and the health care system at large. These three topics were used to structure the results, and are used as headings below. Within these topics, themes and illustrative quotations are presented in italics.

### Ambition to become a family physician

One of the questions asked to FPs was ‘what made you decide to become a family physician?’ All FPs were clear about their initial drive to apply for the MMed-FM. They emphasised that as a medical officer they could develop their affinity for the full spectrum of medical disciplines. One of them describes how the start of the programme came along as a *golden opportunity* for them to continue *to be an all-round doctor, but at a higher level of training* in order to *deliver a more specialised sort of health care to* his *patients.* Another FP said:

(553, man, 1st FP at his hospital & medical superintendent, interview)

"‘To be a senior doctor, someone who is having more training, but still have access to all these people. Then family medicine came and I came to learn that it is actually that, plus management, plus community health. So I think I liked the whole mix of patients. The traversing from one age group to another age group. That is what makes it exciting to me.’"

Another reason to opt for the MMed-FM was the ambition to be valuable to the community. As one FP expressed it:

(032, man, 2nd FP & medical superintendent, interview)

"‘Having worked in various places I knew that this is really the kind of thing that is required if you consider the challenges that we have right now as a country. The kind of doctor that we need to equip so as to meet the challenges that we have right now, I know that family medicine offers a good opportunity to be equipped to deal with the challenges that exist as it is currently, that we are facing.’"

This aspect also became evident when some of the FPs stated what prevented them from leaving the public sector to join private health care. Although FPs recognised certain advantages of working in the private sector, such as higher remuneration and greater availability of drugs and diagnostic tools, they felt that in the public sector, serving the poorer segment of the population would increase both their impact and their emotional rewards:

(047, man, posted five weeks before as 2nd FP, interview)

"‘The people need us and I think you cannot just neglect everything for the sake of money. There are people that will come and say ‘thank you’ and that is more than 100,000 shillings. Just a thank you is enough, because you made a difference. I want to make a difference. I really want to make a difference.’"

### Roles perceived for and fulfilled by family physicians

The most extensive descriptions of what being a family physician entails were found in the documents on which the family medicine specialism is founded. The policy offers the following definition:

"‘The Family Physician is a medical doctor providing competent and comprehensive clinical care over a wide range of patient conditions, considering the patient’s physiologic, psychological, socio-economic, cultural and spiritual dimensions within the context of their family and community, and not limited by the person’s age, gender, organ system or disease entity. Technically, the Family Physician is distinct from other specialists by being a “generalist”, with a “working knowledge’ of other specialties able to offer comprehensive and quality care.’"

Colleagues had difficulties identifying specific tasks assigned to FPs. *Being a comprehensive care provider* was the most commonly acknowledged role. Answers to the question ‘What is family medicine?’ would commonly focus on the aspect of FPs providing comprehensive care. FPs particularly mentioned the satisfaction they got from not having to turn away patients with particular conditions, as a result of their broader skills. Colleagues were also especially delighted about having a generalist consultant in their hospital, as was reported by a nurse:

(371, woman, nurse, FGD)

"‘A family physician […], this person is able to go around the person, or even the family. He can tackle the child [illnesses] in that family. He can tackle the mother who has gynaecology issues. He can touch the father who has an issue even with marriage, because he has done psychology as well. Our family medicine Dr has done even psychology, so he can go around the family issues. He can give a health talk, so you will not be channelled to one place […] like a surgeon who is only for bones and surgery and so on and knives. [Other nurse agrees with “and knives”] If you give him a case of delivery he wouldn’t deliver, but [the family physician] will go to maternity and deliver the baby very well, do maternal issues which is complicated and he can come out well.’"

Apart from being a generalist, the aspect of *providing longitudinal care* was mentioned. One FP described the aspect of longitudinal care as follows:

(861, man, 1st FP & medical superintendent, interview)

"‘You know if you are a surgeon […] you remove the cyst and that’s all. But for family medicine we have this longitudinal relationship that we have with our patients. We want to see patients and see them over a long period of time. We want them to call us, have a discussion with us, have faith in us. That is the kind of doctor that the population requires.’"

Most of the FPs felt a strong need to establish *links with the community*:

(237, man, 1st FP & medical superintendent, interview)

"‘In some hospitals the wards are full. Many many children sick and all that. They are not being reversed easily by just being, sticking there in the hospital and working very hard. You must confront a problem at community level.’"

Some of them felt they had to *go into the community* themselves, while others intended to detect and solve issues emerging in the community through the community health units which are currently being established:

(441, man, 1st FP, joined by 2nd FP five weeks before, interview)

"‘The role of the family physician is to provide leadership […] to the primary care team. […] The primary care team includes the clinical officers, the nurses, the social worker, the nutritionist, the public health officer, the community health extension worker, who usually is a public health officer, and a community health worker. So the family physician is expected to be the lead person in providing guidance to that team in overseeing patient care as the front-line team for the hospitals for that community.’"

Colleagues of an FP described how *he pushes patients from* the *community to the hospital*:

(290, woman, nurse, FGD)

"‘I have seen him pushing patients from the community to the hospital. Like me I’m in OPD. Like he comes with a patient, he tells me that this patient is having maybe fibroids which are very big but the patient maybe has been at home. He has collected the patient from home to the hospital and he does a hysterectomy here. So about what I have seen with our family doctor here is that he goes beyond the hospital and he pushes patients from the community to here. But I cannot tell really how he does it, but I see him coming with patients from community to here.’"

To one FP, the community aspect was in fact the essence of family medicine:

(441, man, 1st FP, joined by 2nd FP five weeks before, interview)

"‘Family medicine is the doctor who cares for individuals as members of a family who belong to the community. The essence is to look at a patient with a reference to the problem that brought them to the hospital in respect to the public they came from and if that problem spills over into the community.’"

Another widely acknowledged role for FPs was *being a teacher.* The policy mentions that FPs have to *[t]each effectively members of the health care team, the patient and the community.* One aspect of it is that the FP is a life-long learner, as expressed by the curriculum:

### ‘Questions and learning opportunities prompted from patient care are pursued using self-directed, life-long learning skills’

The strategy explains this more clearly by adding that this *Life-long Learner* is *responsible for organising Continuous Professional Development (CPD)* as well as *Continuous Medical Education (CME).* One nurse described how the FP not just enhanced her medical knowledge, but also encouraged her to provide care beyond the medical problem:

(290, woman, nurse, FGD)

"‘We hold our CME’s here […] but it is also good that when he finds you in the department he is also good in giving health education. Maybe not necessarily concerning medical work. He is also social. He comes, he enlightens you and he advises you. You are not maybe supposed to deal only on nursing profession. It is good to do something else beyond nursing.’"

The key documents also identify a managerial role for the FPs. The strategy refers to the FP as *a leader,* or a *decision-maker and where possible a change agent in hospital administration and resource management within his area of work.* The managerial role became more evident from the interviews with those FPs who were posted as medical superintendents:

(861, man, 1st FP & medical superintendent, interview)

"‘[Since] I got medical officers I have moved mainly into the role of medical superintendent, which involves managing finances, managing schedules for doctors and managing patients and changing systems, the ones that I see are not working properly. Then I change so that they work, they work properly, so some of them now see me more in terms of my role as a manager for the hospital or the leader for the hospital.’"

Some FPs were actually *agents of change,* finding themselves posted as medical superintendents in run-down institutions. Two FPs, both having been instated as medical superintendents about six months earlier, described how they had been able to double the revenues of their hospitals. One of them gratefully took the opportunity to ‘restore law and order’. Within one week he was able to send out three ambulances, which had been kept in a garage by a local authority for the past year, to rural health facilities. Other urgent issues were also referred to:

(237, man, 1st FP & medical superintendent, posted six months before, interview)

"‘When I came in there were serious organisational problems […] The fact that the health care providers were not coming everyday to see patients before I came. And they were not doing ward rounds, clinics routinely but they are doing it now everyday of the week. There were no continuous medical education whatever going on. And of course we had not computerised some of the basic areas like the drug dispensing areas and the money collection areas. And as a result a lot of corruption and a lot of money was being lost. And in the last six months as we have seen these loopholes and computerised the systems you can see there is a difference because we were collecting only half a million shillings and now we collect 1.2 million shillings a month.’"

FPs who are not posted as medical superintendents can still acquire the momentum to pursue change, though this depends on the willingness of the leadership:

(441, man, 1st FP, joined by 2nd FP five weeks ago, interview)

"‘There are substantial challenges in trying to fit into an already conceived and organised hierarchical order. And the family physicians entering into a hierarchical order which is perceived to be orderly, with new ideas, sometimes is not necessarily taken kindly. Luckily in this hospital I think the leadership has been very foresight and interested in any changes that would eventually spill of to assist in the community. So it has actually been very […] easy to fit the concepts into the institution, because of the support by the administrator, especially the medical superintendent.’"

Another role assigned to FPs is that of doing *research.* The policy asks FPs to *provide a forum to discuss a common research agenda for Family Medicine.* The strategy sees the FP as [*a*] *researcher, participating in a discipline initiated, practice-based and outcome oriented (interdisciplinary) research agenda, that is critical to a long-term specialty and district level clinical improvements.* Hence research is described not just as a way of favourably influencing the medical setting locally, but indeed to make family medicine itself viable as a new specialty. However, little research was performed by FPs. So far, one FP had engaged in research:

(237, man, 1st FP & medical superintendent, interview)

"‘Oh yes we are involved in research. Especially in the comprehensive care clinic. We have done quite a lot of research work there on home-based care of patients with HIV. We have done a lot of research on uptake of visual inspection of patients, comprehensive screening of patients with HIV for cancer of the cervix especially the women. We have been doing a pilot study here on how effective early diagnosis of cancer for the cervix would help to change the life of these women.’"

Yet the other FPs also intended to conduct research:

(509, man, posted two weeks before as 1st FP, interview)

"‘Research will be ongoing on our parts as clinicians. That is one thing that we have learnt when we were in school: we need to be involved in research, quality improvement projects, so that you can impact change. Nowadays people want to see evidence. Everything is evidence based. Not just into intrusions, anecdotal sayings of evidence, but people want evidence.’"

(047, man, posted five weeks before as 2nd FP, interview)

"‘I have been thinking about [doing research] this morning. […] I have just been looking at the number of patients with portal hypertension, that is haemorrhage, and it is overwhelming. I think it would be important maybe to look at such issues in the future. Of course as we settle I think there are many more things that we can look into. So research is definitely in the back of my mind.’"

Reasons given for not doing research were a perceived lack of funds, time, tools and information, and inadequate training and supervision. Apart from conducting research to develop the setting for family medicine, *establishing family medicine* as such was seen as one of the roles for FPs.

(237, man, 1st FP & medical superintendent, interview)

"‘I plan at least to make sure that I establish a system of health care in this county […] as an example to the others. Show how a family physician can change his catchment population for the better. So in the next 3 years I would like to make much more use of the opportunity I have here to change the whole place. I’m not here permanently, but during the time that I am here I want to leave what we call a legacy. A legacy of family medicine. How the whole community changed. How hope came in and then after that I would like to go to another place where they have a problem like what I found here and change it.’"

### Challenges

As the FPs indicated the roles which they perceived themselves as carrying out, they also identified the challenges that arose while performing these roles. Our focus was on challenges directly affecting FPs and the discipline of family medicine. One regularly recurring theme was that FPs felt they had been posted to *fill human resource gaps.* This effectively forced them to take up the role of one of the other specialists, preventing them from fulfilling their family medicine mandate, whereas the strategy actually states:

"‘The Family Physician is the person, together with the [District Manager Of Health], responsible for the introduction and use of the Kenyan Essential Package of Health (KEPH) in his catchment area. They should therefore not be seen as a replacement of a specialist, but rather as the first contact physician, referring – when appropriate – to other specialists, working as a team.’"

The actual experience was often that they were indeed *a replacement of a specialist.* One FP brought in the concept of misuse:

(553, man, 1st FP & medical superintendent, interview)

"‘[A]s a family physician I see all sorts of patients. I don’t replace a paediatrician. I don’t replace a physician. But I see adults and I see children. But like in a posting there is some, at some level they think that now that we have a family physician we don’t need a paediatrician, we don’t need a physician, which is not true. That is what I say misuse. So you are now sent to a facility to act as a paediatrician or as a physician or an obstetrician and when the obstetrician is not there, then you are the obstetrician. When the obstetrician comes back, then you are completely withdrawn from that site. […] Whereas it actually should be that I am everywhere yes, but […] when the obstetrician is there, I am still there. […] And it doesn’t mean that when he comes, I should move out. It is not like he takes my place in times. My roles are different from his roles.’"

Another FP explained how being posted to fill human resource gaps prevented him from following up on his mandate as an FP, although this improved over time as more consultants were brought in:

(441, man, 1st FP, joined by 2nd FP five weeks ago, interview)

"‘The actual major challenge has been the clear allocation of your assignment as the family physician. My assignment was such that initially I ran paediatrics, I ran internal medicine, I ran OPD casualty. On top of that [I had] to start a family medicine practice in the hospital. So I think since the family physician is trained comprehensively, when there is a gap or lack of another physician the family physician is […] requested to fill that gap. So essentially it over burdens the family physician and he finds it sometimes difficult to realise the starting of a department which is running efficiently. That has been one, that is a major challenge.’"

To ensure that FPs are able to perform the roles they have been trained for, this FP proposed that *the ministry itself* should give *very clear directions to the institutions about the posting of the family physicians* and their assignments. It would also be preferable if FPs were only posted to hospitals where the other departments already had appropriate consultants. A solution suggested by another FP was to always post FPs in couples, as was strongly recommended in the strategy. One FP actually understood his task as filling gaps:

(509, man, posted two weeks before as 1st FP, interview)

"‘In essence family physicians are there to fill that gap which has […] for a long time been felt in the health care system. But probably in a district hospital like this you may only have a surgeon, so anything that comes that is paediatric related or internal medicine related is referred to the province. And we are here to fill that gap.’"

As one of the reasons why they are being posted to fill human resource gaps, FPs mentioned *lack of awareness of family medicine.* Once posted, FPs find themselves explaining their roles to their colleagues and the community. Several noted that family medicine was not sufficiently marketed and that their job assignments would be made easier if their colleagues had been sensitised about their roles before their arrival. One FP who had recently arrived to take up his posting gave an example of how the lack of awareness of his roles led to inefficient health care provision to a patient:

(509, man, posted two weeks before as 1st FP, interview)

"‘Just today I was quarrelling with one of the interns for referring a patient without my authority. Because she had a condition she thought, and after she discussed with one of the medical officers they thought it cannot be handled [in this hospital]. We had not fully investigated the patient so we were actually referring a patient that we didn’t know what we were referring.’"

Colleagues of the FPs acknowledged the FPs’ ability to provide comprehensive care, although they were unable to define what exactly should be their clinical niche. In one focus group discussion, the attending nurse and clinical officer assumed that FPs were in essence trained to treat chronic illnesses in patients from all age groups. Hence, they would not refer patients needing acute care to the FP, while in fact he had been trained to treat these patients as well. Some colleagues were able to recognise the FP’s teaching, community and management roles. They acknowledged their lack of awareness themselves:

(518, man, clinical officer, FGD)

"‘I know we really don’t have much that is known about family medicine in Kenya, to the general population and even the medical […] because it is a very new course. We really don’t know what is the job’s specifications, we don’t know how far they should go, how they should run. They are not in the plain media as we know surgeons and orthopaedic surgeons and medical… Let’s say internal medicine is what we would call […] a family physician in Kenya, most of us. So that to my realisation is that family medicine is quite open and does a wider scope than most of these other courses.’"

On every occasion, the colleagues were eager to get to know more about family medicine. When asked whether improvements had been made since the FP had been posted, they would mention that the FP had attracted more health professionals, which reduced the workload, that clinical gains had been made and that organisational issues had been addressed:

(981, man, lab technician, FGD)

"‘There has been quite an improvement in issues of maternal care. Especially surgeries of the mothers. Some of them used to be referred to [a town nearby] and other hospitals, but when he came, especially when he is around, he is able to take care of all the patients […]. He is able to handle them well. He is able to do surgeries, caesarean sections, and he is also a good manager. […] Although [it] is not his speciality he is doing pretty well, and he follows issues to the letter.”"

In all, FPs would like their colleagues to know what their mandate is, and at the same time, colleagues would like to know what exactly FPs are expected to do:

(624, man, medical officer intern, FGD)

"‘My expectation is that there will be a demarcation in terms of their work and the work of other consultants. It should be clear cut, so that we benefit from all of them. So maybe if the department will strengthen them in such a way that we know when to refer patients there and to other particular people who have been trained by the family physicians to work in that particular setup, that would be my expectation. Because as it is right now their work is integrated with the rest, so there is usually confusion as to what the family physician does and what the [other consultants do]. So if there was a clear-cut on this is the department of family medicine and these are the cases that they should deal with it would be a little better.’"

It is also pivotal to the establishment of family medicine that medical officers are informed of the possibility of becoming an FP. A medical officer intern from Moi University had met one registrar of family medicine, but hardly knew what it entailed. His fellow medical officer intern from Nairobi University had only heard about the existence of this specialty in Kenya upon arrival at this particular hospital. The expected initiation of the post-graduate training in family medicine at both Nairobi University and Aga Khan University is likely to improve this situation. Nevertheless, the FPs urged that current undergraduate students be sensitised about their specialty, and that family medicine be incorporated into their rotations as well. One of the reasons why FPs would appreciate such measures is the *resistance from colleagues* they face. Apart from the fact that unawareness itself induces resistance, it partly stems from colleagues’ perception that a consultant who can cope with many clinical fields might render them superfluous:

(047, man, posted five weeks before as 2nd FP, interview)

"‘The issue is, for a long time there have been recognised specialities, and when you bring something new it is always a challenge, where there are deeply rooted ideas. And then to make matters a bit more complicated we deal with very many fields of medicine. We are trained in paediatrics, we are trained in medicine, we are trained in surgery, trained in [obstetrics and gynaecology], trained in psychiatry, we have a bit of ear nose throat, a bit ophthalmology. So of course people will perceive that as a threat. Because if I am able to do, to act as an internist, then there will be no work for the internist. If I am able to do most of the surgeries that an obstetrician can do, then there will be no work for the obstetrician. It is viewed as a threat.’"

Another cause of resistance among colleagues is the common phenomenon that people who have been doing things in a particular way for years are sometimes reluctant to accept attempts to change the status quo:

(861, man, 1st FP & medical superintendent, interview)

"‘The flow of patients is not very good. The hospital is spread all over. The pharmacy is in one corner, the records is in the other corner, and the clinicians are in some other corner. So it becomes, it is a bit confused, okay. […] I was trying to restructure so that I have patients report to records at the first room, […] then the payment point, then the clinician, then the other sections. But I encountered a lot of resistance. Resistance from staff. They say “no, we have always worked like this and why do you want to change?” you know. But for me I was seeing like there is a problem in patient flow. And so I have had to shelve that plan for the time being.’"

Family physicians often felt *overwhelmed* by their workload, and both FPs and their colleagues generally assumed that this sense of being overwhelmed was due to the many roles their mandate encompasses:

(518, man, clinical officer, FGD)

"‘[The FP’s] availability in this hospital is quite little. I know he handles, being a medical superintendent and maybe […] the only doctor with a Masters degree in the whole district, has a lot to handle. So he sees very few patients. […] According to the government he is supposed to go to other health centres and district dispensaries, get patients from there and bring them to the hospital. But being the only person, being the only person in the management and being the supreme, he cannot be able to do the management, go to the health centre, go to other dispensaries.’"

Despite being trained to do home visits and prevent diseases in the community, several FPs acknowledged that the closest they got to the community was the door of the out-patient unit. In this respect, one FP described his work as fighting fires:

(032, man, 2nd FP & medical superintendent, interview)

"‘There is what I think would be the critical numbers that you need to run effectively, to have a personal relationship with a patient on a long-term so to be able to offer longitudinal care. So the challenge right now is that you have so many patients. And when you have so many patients, one is that it reduces the contact time with the patients. You are forced sometimes to clear the queue. You know you have 50 patients outside and you have a short time. So really being able to see a patient as comprehensively as you would like to is a challenge. Again because of the numbers. The patients are so many, the people to see them are very few. So it becomes like you know when there is a fire you go fight the fire. And when that fire is done another fire lights somewhere else, you rush there. So when you keep running you can’t really do whatever you really want to do. So the volumes […] of the patients is as the, you know what you can do as an individual is a challenge for a family physician.’"

Another FP noted that he was uncomfortable with the fact that if he left, no other consultant would be there to provide care to the patients:

(553, man, 1st FP & medical superintendent, interview)

"‘Satisfaction in my job. Satisfaction in the sense that I don’t want in the future to be in a place where every other day I’m going to be feeling guilty that I haven’t done something. That I’m going to be feeling guilty that I ought to have done something differently […]. And the kind of situation is there, […] you actually need somebody else to make you know that you are doing well. Because if you are the only doctor and then something bad happens, you can think that it is because of you. But it is because you are the only doctor. You know you can’t be in the same place 24-hours, or all the time. So I look forward to be working in a situation where I have my input to the maximum that I can, and when I have gotten to that level somebody else does the rest, […] so I’m not going to feel… I have satisfaction that I have done and helped and things do not go bad when I left.’"

FPs who were posted alone looked forward to the posting of a second FP in their hospital. They assumed that they would then be able to carry out all their roles as a team:

(861, man, 1st FP & medical superintendent, interview)

"‘I have not been able to do my other roles of going to the community for outreach because then I will just stretch myself and I can’t be able to do that. And I am looking forward to, there is actually a new family doctor who is posted. I am hoping that when he comes when he eventually starts work, then he will be able to do now the community outreach and complement me on that aspect.’"

The sense of being overwhelmed was not confined to FPs only, but felt throughout all cadres of medical personnel. Several of them pointed out that the number of doctors had increased, diminishing the burden considerably. This was particularly true if the hospital had been transformed to a training site for clinical officers, medical officers and sometimes also FPs, as the influx of both consultants and internees came as a relief. FPs and their colleagues acknowledged that the lack of doctors was currently not as severe as the lack of nurses. One FP was involved in holding the interviews for nurses employed by the Ministry of Medical Services. One complaint he had was that he did not have the authority not decide which particular nurse would be posted to his hospital. Another issue was that medical superintendents were unable to dismiss poorly functioning employees. Several FPs mentioned that they had tried to get particular employees dismissed, but were hampered by the bureaucracy involved. As a result, their hospital was forced to continue working with staff which did not perform their job adequately. Efficiency was also compromised by a lack of anaesthetists and supporting staff. On one occasion, the investigator observed how an FP who was the medical superintendent ordered all surgical cases to be referred to a town nearby, since the anaesthetist was unable to attend due to illness. On another occasion, another FP functioning as medical superintendent explained that he had managed to build one more operating theatre, but still only had one anaesthetist. In order to use both theatres at the same time, the hospital now depended on his own skills as an anaesthetist.

Other perceived challenges included *financial* and *infrastructural* issues. At a personal level, health care workers of all cadres found themselves inappropriately remunerated. At a higher level, FPs were dissatisfied with the government’s decision not to adhere to the Abuja Declaration, which stipulates that Sub-Saharan African governments should invest at least 15% of their gross domestic product in health care. FPs said that if this minimum were adhered to, the issue of insufficient supplies could be solved. In the current situation, the costs incurred to overcome shortages of drugs and diagnostic tools are covered by generally impoverished patients. FPs expected that this was causing delays as patients decided to wait before seeking health care for financial reasons, leading to worse conditions and higher costs. Even if patients do seek health care, lack of supplies still causes delays when patients have to travel to a clinic or pharmacy in another town to buy appropriate drugs or tests, or get a blood transfusion:

(032, man, 2nd FP & medical superintendent, interview)

"‘You want a transfusion but there is no blood. There is no blood and the patient is dying. So you end up referring the patient. Of course at the end of the day the cost of referring is higher than the drug that you wanted to buy.’"

One FP showed that securing the availability of supplies itself is a way to increase revenue:

(861, man, 1st FP & medical superintendent, interview)

"‘We realised the laboratory department was not getting the support. They were not getting reagents, there were broken equipments. So we decided to put aside money to repair the broken equipment and also to supply reagents. And I mean ensure that the supplies, the reagents that are required to carry out tests are available, because previously people would come and we don’t have this. When we did that, and I have evidence, we were able to increase… What the laboratory department would bring in a month was 30,000, but we have actually tripled just by ensuring that the supplies are there. So now in the last month we collected about 110,000 from that department.’"

An organisational issue impacting on the ability to repair equipment and on the supply of drugs and diagnostic tools was the tendering system currently used. Every service that could possibly be required during the upcoming year has to be tendered in the national newspaper Daily Nation. The company making the best offer has to be contracted, even if it is based in some distant location. In practice, this makes peripheral hospitals dependent on supplies and services delivered from Nairobi, adding to delays:

(032, man, 2nd FP & medical superintendent, interview)

"‘There is something called bureaucracy: if I ran out of a drug and I have the money I can’t just go and buy even if it’s next door. Because government rules require that you must have a contracted supplier that has been tendered and tenders open competitively. […] So if somebody from Nairobi, the big city, wins a tender and the same drug is in [this town], I will not buy it from [this town]. I have to ask the contracted supplier in Nairobi to send it. So if you need it for a patient who is there, maybe it will take a week to come.’"

This FP added that all expenditures had to be authorised by the district’s treasury department, for whom *‘issues of emergency are not necessarily their priority’*. Another issue several medical superintendents faced was *being surprised by debts* from the past:

(861, man, 1st FP & medical superintendent, interview)

"‘When I came over I expected for example to be handed over and be told this is the amount of debt. I expected to find a certain system working. I did not find that so I had to start creating the systems as I would want them to be. […] I found a system where people would just collect the money from patients and use without banking and planning for that money. So I found very huge debts and I am sure you saw one of them telling me I supplied things in 2008 and I have not been paid. Now nobody told me for example that this lady has not been paid. But now I need to handle and that’s why I was telling her “please if you can prove that you actually supplied goods to this hospital in 2008 we will find a mechanism of paying you”. […] So I keep running into those and that is affecting my planning and delivery of health care generally. Because when I do the budget, I budget for food for example and say I’m going to use 200,000 for food. Then now I have to pay 300,000 debts. See that will affect the quality of food that I will give to my patients.’"

One FP mentioned that the MMed-FM training programme had suffered from a *lack of standards.* Although other FPs did not literally mention a lack of standards, this lack became clear when comparing their contradictory responses to a question about shortcomings of their training programme. While one would state that the training had underemphasised management, another would mention that the focus had been too much on internal medicine, while yet another felt that it had focused too much on the clinical aspect, leaving too little room for the community aspect:

(509, man, posted two weeks before as 1st FP, interview)

"‘[The] administrative capability during my training was not given much impact. That is unfortunate because as you come out you are expected to be an administrator in one way. And yet you probably took a very short time with a medical superintendent role or any administrator of any place. So you come out feeling that deficiency and you have to have probably an on top training when you are already working.’"

(047, man, posted five weeks before as 2nd FP, interview)

"‘Inasmuch as we are supposed to be experts in history taking and all that, I tended to think maybe we are putting a bit too much emphasis on that. It is important, but you cannot put the emphasis on that at the expense of some other things. The training of family medicine in Kenya at present tends to focus more on the internal medicine aspects of things, and maybe at the cost of others. Because we need surgical skills as well. We need skills in acute care. But you find sometimes…, initially we tended to concentrate more on the medicine part and to some extent neglect the other disciplines.’"

(237, man, 1st FP & medical superintendent, interview)

"‘Some of my teachers were completely clinically oriented. Especially the ones from the USA, which was good. They were very strict on that and we needed to acquire that skill. However there was a tendency to be biased and to look like the community activities were inferior. I could see it, and it worried me a lot. Because I was fearing that the final product, the person who might come out, might actually be a person who will not be willing to work in the community, if he is encouraged to stick in the hospital areas to wait for problems to be brought to [him].’"

FPs particularly mentioned that some of the registrars had not been stimulated to develop a community intervention project, even though this was one of the requirements of the programme. The FPs pointed out the risk that this could lead to FPs who did not actually appreciate engaging with problems in the community. Some FPs felt there was a certain tension between the American and Dutch teachers, with the Dutch being more focused on the community aspect, while the American put more emphasis on the clinical skills:

(237, man, 1st FP & medical superintendent, interview)

"‘I also noticed during my training that some of my teachers were oriented more on hospital-based care. They put a lot of emphasis on the clinical skills, attendance to patients and all that. However there were teachers, especially the ones who came from the Dutch, [who] had an orientation and emphasis on community. I could see as a student that it was a bit of a push and push strategy. Because one person is emphasising these people must work in the wards and do everything there, which is okay. […] Another person was realising that the wards will never be empty unless we approach it on both sides. Involve the community and do quite a lot.’"

The same FP noted *‘a significant level of intimidation’* from the teachers, and perceived the risk that this would create timid FPs, not daring to change the status quo.

Apart from being too overwhelmed with clinical or managerial tasks to do research, another issue was that FPs felt that they had not received sufficient training to do research. Several FPs mentioned that this was a result of the programme’s dependence on both the School of Medicine and the School of Public Health. The School of Public Health was supposed to provide lectures on research methods and statistics, and lectures had often been cancelled:

(441, man, 1st FP, joined by 2nd FP five weeks ago, interview)

"‘The registrars are dependent so much in the master of medicine training on the School of Public Health, whereas they are admitted in the School of Medicine. […] I think the courses that are trained, are taught by the School of Public health, the family physicians should be the people who should be inducted to join the university to train those courses. […] Because the School of Public Health says that you come this month, but then after a short while they change [and say] “come next, after two months”. Then after those two months they say “no, we will not do it now”. So it becomes a real challenge. I think the School of Medicine and specifically the division of family medicine should be trying to build capacity using family physicians to be able to run this training without depending on the School of Public Health.’"

Even though serving the underprivileged was seen by some as a reason to become an FP, it could also be seen as a challenge. Two emerging aspects were that *serving the impoverished* led to great financial constraints, which was made even worse by patients delaying to seek health care:

(553, man, 1st FP & medical superintendent, interview)

"‘I would feel a little guilty about the charge which is difficult in certain places. Because I still think of what the patient is going to do. If the patient doesn’t come to me it still bothers me what the patient is going to do out there. Because one of the roles of a family physician is to ensure that patients dwell in the community. So if the patient isn’t coming to me, it bothers me that the patient is not coming to me. So it is because of finances that I would adjust the cost that I’m charging. So finances are major.’"

Another issue leading to patients arriving late in the health care system, resulting in increased complications, was that they first tended to seek advice from other health service providing entities:

(047, man, posted five weeks before as 2nd FP, interview)

"‘You know if you look at something like epilepsy or convulsive disorders, people believe they are caused by evil spirits. So they never come to the hospital. They go to prayers. They go to witchdoctors and many other places.’"

One challenge particularly for medical superintendents was that they hardly had any influence on the staffing of the hospital they worked in. As a result of staff being hired by the ministry, *dysfunctional staff* cannot be easily dismissed. Hence these staff members continue to hamper the team of health care providers. One FP claimed to have developed sufficiently strong ties with the governing bodies to be able to dismiss employees through them, preventing such issues from arising. The government attempts to provide the hospital with local staff, but in some places local staff is hardly available, leaving little choice as to who to employ, and leading to a higher proportion of dysfunctional staff. Another problem is that FPs depend on two ministries. The Ministry of Health Services governs the district, provincial and national hospitals, while the Ministry of Public Health and Sanitation takes care of the dispensaries, health centres and the community. One FP explained that it was important to ensure that he was able to influence both ministries in his district, as his success was irrevocably linked to their performance:

(237, man, 1st FP & medical superintendent, interview)

"‘We have two ministries within the health sector in this country. We have public health and sanitation. We also have medical services. Medical services has been concentrating a lot on hospital care like what we’re doing here, while public health has been concentrating on the health centres and the community. I have made an observation that whenever there’s a problem with the health care systems in the community and in the lower-level facilities, those problems will be shifted to this hospital. And therefore it has been a challenge trying to ensure whatever the other ministry is doing I’m very much involved and very much aware. Because when they go down they bring me down. When they are doing very well I also do very well. Because our patients come from the same communities that public health is supposed to take care of. […] So that was a challenge: how to bring the two together, because I found [the two ministries of health] were totally working each differently and yet they want to achieve the same goal. So I am happy to report that we have, we are now being a team. Thanks to the communication skills I learnt in family medicine.’"

## Discussion

Kenyan family physicians (FPs) had opted to specialise in family medicine believing that this would enable them to be most valuable to the community. In order to achieve this they are trained as generalists, but at a senior level, and their training also includes health care management, teaching and research skills. They perceived their tasks to be just those, though research was not perceived as a major component. Their colleagues agreed with their mandate as a generalist and, to a lesser extent, teaching and management. The clinical roles were the tasks most commonly performed by the FPs, while management was the most common task among FPs posted as medical superintendents. Teaching was emphasised as well, but going into the community and doing research was seen by some as too time-consuming and resource-intensive to carry out. Others saw the community aspect as the essence of their job description. In addition, FPs acted as agents of change, establishing family medicine at their institutions and associated governmental bodies. The main challenges FPs faced were being posted in situations where they were expected to function as just another type of specialist, lack of awareness among colleagues of the roles of an FP, lack of time, lack of funds and inadequate training.

This study investigated family medicine in an African setting and the challenges faced by FPs attempting to establish a new medical discipline within an existing health care system. The study question was kept broad on purpose, as the study was held in the context of a new training programme which had not been evaluated before. Thanks to its timing and comprehensive approach, this study yielded valuable information for policy makers in Kenya and other countries desiring to strengthen or initiate a discipline like family medicine. Qualitative methods are the most suitable to assess the opinions of FPs and their colleagues about family medicine, and their views on the challenges that FPs face. A valuable aspect of this study is that it applied triangulation through the use of an array of methods, including interviews, focus group discussions (FGDs), observations and document analysis. Despite the limited number of eligible FPs in Kenya, data saturation was reached. The preliminary results were presented to the participants during a workshop, enabling them to comment on the information. One limitation of the study was that no fellow consultants of the FPs participated in the FGDs, so we did not assess their possible input or the resistance they might entertain concerning family medicine. Another limiting factor was that three of the eleven participants of the FGDs took a short break during the FGD to take care of other duties. More importantly, the FPs who were observed are likely to have shown different behaviour as a result of the presence of the principal investigator. Although the interviews and FGDs were very carefully transcribed, there were some instances where one or more words could not be identified from the recording.

One of the major challenges for this first group of Kenyan FPs was being posted to fill human resource gaps. Being posted to fill such gaps effectively means that the FP does not practise family medicine, but ends up fulfilling the roles of other specialists. This is likely to threaten their ability to do the two main things they initially intended to do. If an FP is posted to practise as another type of specialist, they will not be able to be as generalist as they would want to. Moreover, the specific patient load assigned to such a misposted FP means that they are likely to have fewer opportunities to serve the community at large, preventing them from effecting the greatest value to the community.

Being posted to fill gaps also led to colleagues of the FPs having difficulties demarcating family medicine as a discipline. One cause of this could be that the current FPs are themselves the persons embodying the concept of family medicine, and if an FP is posted to fill a gap, family medicine may appear to colleagues like just this other specialty the FP ends up practising. This results in unjustified expectations among colleagues and the community, preventing the firm establishment of family medicine in the Kenyan health care sector. Being posted to fill gaps thus hampers colleagues’ awareness of what family medicine is all about. Interestingly, once sensitised, colleagues showed eagerness to learn more about family medicine.

To increase awareness, several FPs came up with the idea that family medicine needed to be marketed more effectively. They called on the government to create more awareness, especially among students preparing to become health professionals and among practising colleagues. An important step would be to ensure that universities providing medical training incorporate courses devoted to family medicine in their undergraduate programmes. This would also sensitise future medical officers about the option of becoming an FP. During our FGDs, it was proposed that other cadres, such as nurses and clinical officers, should get their own post-graduate degree in family medicine. Realisation of this proposal would facilitate FPs fulfilling their mandate in the way it is defined in the government’s family medicine policy.

The role of change agents as taken up by some FPs is likely to be constrained by the illusion that an FP is trained merely to replace another consultant, as well as by a lack of awareness of an FP’s mandate among colleagues. Lack of awareness may lead to more resistance among colleagues whenever an FP attempts to introduce structural changes. This is aggravated by centralised staffing policies, which means that FPs who are posted as medical superintendents are unable to replace members of their health care team if they are dysfunctional. The health care team would become more adaptive if medical superintendents had more power in the process of making decisions about whom to employ in their hospital. However, before more power is handed over to medical superintendents, there must be a way to ensure that these medical superintendents are themselves appropriately appointed. There is anecdotal evidence that medical superintendents have been selected without even being consulted themselves, let alone the health care team they are supposed to manage.

The results show that FPs posted as medical superintendents were able to considerably increase the cost-effectiveness of their institution. Obviously, one aspect enabling them to achieve such major gains is that these FPs seemed to be posted in institutions with the poorest performance. Hence, there were many gains to be made, which they did. Cost-effectiveness will increase further once medical superintendents are given the ability to optimise their health care teams by hiring and firing based on their best insight. Apart from finding alternatives to centralised staffing, the adaptivity of the services provided would be enhanced by replacing the tendering system prevailing in Kenya by a more flexible approach. It has become clear that tendering services to the cheapest provider is a cause of inefficiency, which sometimes, or even frequently, leads to more complications and higher costs. Moreover, the FPs would like the government to adhere to the Abuja Declaration, expecting that a higher national health care budget would enable them to serve the population better.

The FPs mentioned that the closest they got to the community was the door of the outpatient unit. The reasons they gave for their inability to go out into the community were lack of funds, transport facilities and time. Similar reasons were reported by Mash and others in a study on the key principles of family medicine in Sub-Saharan Africa [12]. Some stated that going into the community would not be cost-effective, as they would just reach one community member at a time. Another FP claimed that it would be cost-effective, as the information he gave to one community member was likely to spread out to the majority of the community. The key point was that he himself went to enlighten the community, as his words would have a greater impact than similar words spoken by a nurse or clinical officer.

The perceived ability to do research was low, due to lack of time, training, funds and information. Several strategies could be used to address these issues. First of all, posting FPs in couples instead of alone, as is already being done by the Ministry of Medical Services, would make it more likely that together they would be able to guide their health care team in doing research. In addition, FPs should not be posted to fill human resource gaps, to enable them to pursue their true mandate. To ensure that knowledge on research-related topics is enhanced and kept up to date, CMEs could be organised nationally for the FPs and locally for the health care teams in the hospitals. The financial constraints on undertaking research could be addressed by FPs choosing to focus on low-cost studies. The issue of lack of information has been recognised and addressed internationally. As a result, these days more information than ever is available to researchers in low income countries with an Internet connection, for example through PubMed Central, HINARI and a plethora of Open Access journals. What is more, Open Access journals often waive publication fees for studies done by researchers in low-income countries, lowering the barriers to publication of their results.

The overall quality of the training programme that the FPs had attended was rated to be good, but shortcomings were reported in the areas of research, community aspects, health care management, surgery and internal medicine. Although one can assume a certain universality in this respect, as students will always perceive shortcomings in any programme they are in. The FPs commented that the inadequacy concerning the research aspect was due to insufficient reliability of the contributions to the programme at Moi University that came under the responsibility of the Department of Public Health. FPs suggested that the programme would be better off without the involvement of the School of Public Health. Instead, they would prefer to see graduated FPs taking up this role. As regards the shortcomings in the community aspects of the programme, FPs suggested that instead of having the students devote one to three months entirely to a community intervention course, it would be better to have an ongoing project throughout the post-graduate training.

## Conclusions

Despite the fact that family medicine in Kenya is still in its early stage, it has already had a favourable effect on the Kenyan health care system locally. Whether this impetus will persist depends on the number of FPs being trained in the years to come. The main roles perceived by and for FPs are those of generalist clinician, manager, teacher, life-long learner and, to a lesser extent, researcher. FPs do fulfil these roles, and regularly also roles such as change agents, and, in this first group of FPs, establishers of family medicine. The FP’s niche is supposed to be in the community, but this is not put into practice in most cases. The main challenges hampering FPs in pursuing their mandate are that they are being posted by the authorities to replace other consultants, the lack of awareness among colleagues concerning their roles as FPs and institutionalised barriers such as centralised staffing systems and the tendering system. Achieving the gains for the Kenyan population which family medicine intends to make requires these issues to be addressed. There seems to be a need for FPs, who are scattered over the whole of Kenya, to get organized, so as to have a stronger voice to educate colleagues about the roles of FPs, to market FP as a new specialty in Kenya and to guide policy- and decision-makers. It is important that the challenges FPs face continue to be assessed, to see if they persist. An exploration of the views on family medicine among patients, community members and colleagues who are not in direct contact with FPs might give further insights into what could be done to ensure a firm establishment of this new discipline in the Kenyan health care system.

## Competing interests

GK is employed by MUNDO, which in the past provided funding for the post-graduate programme. PC is a graduate and staff member of the post-graduate programme at Moi University. GD has been teaching in the post-graduate programme.

## Authors’ contributions

CV conceived of the study, helped design it, interviewed the participants, coded the interviews, moderated the FGDs, coded the FGDs, interpreted the data and wrote the manuscript; GK supervised the study, helped conceive of the study, helped design the study and critically revised the manuscript; PC supervised the study, helped design the study and critically revised the manuscript; GD supervised the study, helped design the study and critically revised the manuscript. All authors have read and approved the final manuscript.

## Pre-publication history

The pre-publication history for this paper can be accessed here:

http://www.biomedcentral.com/1471-2296/13/32/prepub

## Supplementary Material

Additional file 1 Questionnaire for semi-structured interviews with family physicians. Questions used for the semi-structured interviews with family physicians Click here for file

Additional file 2 Questionnaire for semi-structured focus group discussions with colleagues of family physicians. Questions used for semi-structured focus group discussions with colleagues of family physicians Click here for file
