# Supplementary material for: What challenges hamper Kenyan family physicians in pursuing their family medicine mandate? A qualitative study among family physicians and their colleagues
Source: BMC Fam Pract. 2012 Apr 26;13:32. doi: 10.1186/1471-2296-13-32 (PMC3418556; doi:10.1186/1471-2296-13-32)
Supplement: Additional file 1 — Questionnaire for semi-structured interviews with family physicians. Questions used for the semi-structured interviews with family physicians [file 1471-2296-13-32-S1.pdf]

Questionnaire for semi-structured interviews with FPs

What is family medicine?

What should a family physician do?

What is the role of a family physician?

What made you choose to become a family physician?

Are your direct colleagues aware of the fact that you are a family physician?

What do you do as a family physician?

What problems occur while performing your job?

Are there financial problems?

Are there organisational problems?

Are there issues which should have been, but have not been taken into account during your training?

What are your plans for the future?

Would you like to work in the private sector?

Would you like to work abroad?
