# Supplementary material for: What challenges hamper Kenyan family physicians in pursuing their family medicine mandate? A qualitative study among family physicians and their colleagues
Source: BMC Fam Pract. 2012 Apr 26;13:32. doi: 10.1186/1471-2296-13-32 (PMC3418556; doi:10.1186/1471-2296-13-32)
Supplement: Additional file 2 — Questionnaire for semi-structured focus group discussions with colleagues of family physicians. Questions used for semi-structured focus group discussions with colleagues of family physicians [file 1471-2296-13-32-S2.pdf]

Questionnaire for semi-structured FGDs with direct colleagues

How is it going in the hospital?

What is family medicine?

What is the role of a family physician?

How differs family medicine from other specialisms?

What do you expect from a family physician?

Does the family physician fulfil your expectations?
